# Implementing Linkage Quality Assessments in Large-Scale Privacy Preserving Infrastructure: Did we kill them a second time and why are there zombies?

**DOI:** 10.23889/ijpds.v9i5.2892

**Published:** 2024-09-18

**Authors:** Kenneth Gersing, Jasmin Phua, Shaun Grannis, Sara Rogovin, Saad Ljazouli, Sam Michael, Chris Beesley

**Affiliations:** 1 National Institutes of Health (NIH); 2 Datavant; 3 Regenstrief Institute; 4 Palantir Technologies

The National Clinical Cohort Collaborative (N3C) uses privacy-preserving record linkage (PPRL) to formulate longitudinal patient histories and for de-duplication across disparate data including electronic health records, administrative claims, and mortality data. The N3C employs a linkage honest broker implementation model which separates the entity holding de-identified PPRL tokens, from the entity holding the substantive data payloads. As a result of this data governance structure, linkage quality assessments are necessarily distributed into 2 distinct processes: 1) Linkage Honest Broker receipt and processing of linkages, 2) Data Aggregation entity assembling data based on received linkage crosswalks. Data is released on a weekly basis.

This presentation will focus on Step 2 for the Mortality data linkages where the downstream data linkage pipelines must assess linkage quality across several dimensions of quality, focused on plausibility. There are 4 types of mortality data spanning the electronic health record, government Social Security Administration death master files, third-party obituary data, and administrative claims. Each of these sources has different levels of authoritativeness and data latency, compounding the challenges of reconciling fact of death including accurate death reporting dates. To address these challenges, we implemented an automated linkage quality pipeline which generates reports that assess linkage plausibility across the various data types, classifying concordance and discordance between data sources including age, dates of death, surfacing plausible and non-plausible activities post-death.

Sharing our real-world implementation will aid conference attendees in understanding how to plan for such activities when considering specific implementation models for privacy-preserving linkages.

